# Evaluating Mechanisms of Postoperative Delirium and Cognitive Dysfunction Following Elective Spine Surgery in Elderly Patients (CONFESS): Protocol for a Prospective Observational Trial

**DOI:** 10.2196/15488

**Published:** 2020-02-13

**Authors:** Jonas Müller, Stephan Nowak, Antje Vogelgesang, Bettina von Sarnowski, Eiko Rathmann, Sein Schmidt, Sebastian Rehberg, Taras Usichenko, Harry Kertscho, Klaus Hahnenkamp, Agnes Flöel, Henry WS Schroeder, Jan-Uwe Müller, Robert Fleischmann

**Affiliations:** 1 Department of Neurosurgery University Medicine Greifswald Greifswald Germany; 2 Department of Neurology University Medicine Greifswald Greifswald Germany; 3 Department of Radiology University Medicine Greifswald Greifswald Germany; 4 Clinical Research Unit Charité Campus Mitte Berlin Institute of Health Berlin Germany; 5 Department of Anesthesiology Protestant Hospital of the Bethel Foundation Bielefeld Germany; 6 Department of Anesthesiology University Medicine Greifswald Greifswald Germany; 7 Center for Neurodegenerative Diseases Greifswald/Rostock Germany

**Keywords:** postoperative delirium, postoperative cognitive dysfunction, spine surgery, neuroinflammation, magnetic resonance imaging, resting-state connectivity, quality of life

## Abstract

**Background:**

Elderly people are at particular high risk for postoperative delirium (POD) following spine surgery, which is associated with longer hospital stays, higher costs, risk for delayed complications, long-term care dependency, and cognitive dysfunction (POCD). It is insufficiently understood which mechanisms and risk factors contribute to the development of POD and POCD following these major but plannable surgeries.

**Objective:**

This study aims to identify modifiable risk factors in spine surgery. A better understanding thereof would help adapt medical management and surgical strategies to individual risk profiles.

**Methods:**

This is a single-center observational study jointly conducted by the departments of neurosurgery, neurology, and anesthesiology at a tertiary care hospital in Germany. All patients aged 60 years and older presenting to the neurosurgery outpatient clinic or ward for elective spine surgery are screened for eligibility. Exclusion criteria include presence of neurodegenerative or history of psychiatric disease and medication with significant central nervous system activity (eg, antidepressants, antipsychotics, sedatives). Surgical and anesthetic procedures including duration of surgery as primary end point of this study are thoroughly documented. All patients are furthermore evaluated for their preoperative cognitive abilities by a number of tests, including the Consortium to Establish a Registry for Alzheimer's Disease Plus test battery. Physical, mental, and social health and well-being are assessed using the Patient-Reported Outcome Measurement Information System Profile 29 and Hospital Anxiety and Depression Scale. Patients additionally receive preoperative cerebrovascular ultrasound and structural and functional brain imaging. The immediate postoperative period includes screening for POD using the Nursing Delirium Screening Scale and validation through Diagnostic and Statistical Manual of Mental Disorders, 5th Edition, criteria. We furthermore investigate markers of (neuro)inflammation (eg, interleukins, C-reactive protein, tumor necrosis factor alpha). Preoperative examinations are repeated 3 months postoperatively to investigate the presence of POCD and its mechanisms. Statistical analyses will compare delirious and nondelirious patients for predictors of immediate (POD) and delayed (POCD) cognitive dysfunction.

**Results:**

This is the first study to prospectively evaluate risk factors for POD and POCD in spine surgery. Recruitment is ongoing, and data collection is estimated to be finished with the inclusion of 200 patients by mid-2020.

**Conclusions:**

The identification of mechanisms, possibly common, underlying POD and POCD would be a major step toward defining effective interventional strategies early in or even before the postoperative period, including the adaptation of surgical strategies to individual risk profiles.

**Trial Registration:**

ClinicalTrials.gov NCT03486288; https://clinicaltrials.gov/ct2/show/NCT03486288

## Introduction

It is well established that the proportion of elderly people continues to grow at an unprecedented rate in western societies [1]. Older patients are at increased risk for an episode of delirium following major surgery, but the rate of complex interventions such as spine surgery in this population is rising [2,3]. Notably, the increase of anterior cervical fusion procedures is three times greater than that of general surgery in this population based on the National Hospital Discharge Survey from 1990 to 2004 [4]. Other procedures including lumbar fusion, laminectomy, and discectomy exhibit an ongoing and similar progression [3,5].

Postoperative delirium (POD) typically evolves within 72 hours following surgery and is defined by the *Diagnostic and Statistical Manual of Mental Disorders, Fifth Edition* (DSM-5) as a disturbance in attention and awareness that develops over a short period of time, fluctuates, and is accompanied by a change in cognition [6,7]. It is associated with increased complication rates, nursing times per patient, length of hospital stay, per-day hospital costs, and 1-year health care costs [8-10]. While the full pathophysiology of POD remains to be elucidated, current literature suggests an underlying multicausal model that includes neuroinflammation, brain network dysfunction, endocrine stress response, and neurotransmitter imbalance [11-15]. POD was long considered a reversible condition, but it is now established that affected patients do not return to their prior quality of life and employment [16-18]. Elderly patients are additionally affected by postoperative cognitive dysfunction (POCD) that persists in about 30% to 50% of cases after resolution of POD or develops independently up to 3 months following surgery [7,19,20]. While POCD can develop in the absence of POD, more severe POD increases the likelihood of POCD indicating that both entities share at least some underlying mechanisms [21,22]. Supporting the idea of shared mechanisms, POD and POCD have both been shown to accelerate the rate of cognitive decline and increase the risk of long-term mild cognitive impairment or dementia, which may ultimately lead to long-term care dependency and institutionalization [20,22-26].

Knowledge of risk factors for POD and POCD, particularly modifiable risk factors, is therefore imperative to enhance informed patient consent, adjust anesthetic and surgical strategies to individual risk profiles, and facilitate appropriate postoperative monitoring [27]. Numerous prediction models have been developed to identify patients at risk, yet recent studies highlight that a general application of these models in clinical routine is limited, not least because trajectories of cognitive decline are not independent of the type of surgery [22,26,28,29]. For example, patients who exhibited POCD following cardiac surgery improved cognitive function after 1 year compared with their baseline level, which contradicts results from mixed surgical populations [26,29]. Differences in preoperative cognitive function and mechanisms underlying cognitive dysfunction possibly resolve some of the discrepancy, which highlights that surgical type-specific studies are required to identify mechanisms of POD and POCD unique to these procedures [7,22,28,30].

Five prospective studies evaluated POD following spine surgery and were unable to identify modifiable risk factors other than intraoperative hypotension [31-35]. Retrospective and secondary outcome analyses suggest that less complex and shorter interventions such as simple decompressions could be associated with lower POD and complication rates compared with complex fusion and instrumentation procedures, rendering the surgical intervention itself a potentially modifiable risk factor [34,36].

In this study, we thus investigate the primary hypothesis that the duration of spine surgery is a predictor of POD incidence in spinal surgery, which was not previously tested as a primary end point in a prospective and sufficiently powered study. Evidence in favor of our hypothesis would justify adaptation of surgical interventions to individual risk profiles as a viable means to reduce the incidence and sequelae of POD without withholding necessary surgery from affected elderly patients. This study will also evaluate the relationship between POD and POCD in spine surgery, which has not been done before but was declared one of the most relevant study areas in a recently published multinational and interprofessional delirium research agenda [37]. Additional end points include long-term cognitive function, quality of life, activities of daily living, mood, and frailty. Underlying pathophysiological mechanisms will be investigated through ultrasound of the cerebral vasculature, structural and resting-state functional magnetic resonance imaging (sMRI, rs-fMRI), markers of (neuro)inflammation, and metabolomics.

## Methods

### Setting and Registration

The Cognitive Dysfunction Following Elective Spine Surgery in Elderly Patients (CONFESS) study is a prospective single-center observational study jointly conducted by the Department of Neurosurgery and Neurology in cooperation with the Department of Anesthesiology at the University Hospital Greifswald, Germany, a 950-bed tertiary care hospital. The trial was approved by the institutional review board of the University of Greifswald (BB 192/17) and registered at ClinicalTrials.gov [NCT03486288]. The Standard Protocol Items: Recommendations for Interventional Trials (SPIRIT) checklist is provided as Multimedia Appendix 1.

### Patient Recruitment and Study Design

Patient recruitment began in February 2018, and the study continues enrolling patients presenting to the Department of Neurosurgery for elective spine surgery. All patients seen in neurosurgery outpatient clinics or inpatient wards are screened for eligibility. Patients can be enrolled if they are at least aged 60 years, scheduled for elective spine surgery without opening the dura, can give informed consent themselves, and are German native speakers. Exclusion criteria comprise any diagnosis of dementia or neurodegenerative disease, psychiatric disease, prescription of central nervous system–active medication (eg, antidepressants, antipsychotics, sedatives, alpha-1-receptor antagonists), inability to participate in follow-up, participation in an interventional trial, electronic or displaceable metallic implants, or active neoplasms. Informed consent to participate can only be given by the patient themself. All baseline examinations are scheduled within 14 days prior to surgery (V0). The day of surgery (V1) includes documentation of routine procedures and a close follow-up of patients in the postanesthesia care unit (PACU) for at least 2 hours or longer depending on the clinical situation. Patients are afterward routinely transferred to the neurosurgical ward or may occasionally require intermediate/intensive care treatment. Postoperative visits (V2) continue for at least 72 hours postoperatively and include detailed documentation of primary and secondary end points. If patients develop POD within 72 hours, daily follow-ups continue until no signs of POD are documented over a period of 24 hours or the patient is discharged (eg, for rehabilitation). Patients are routinely seen in the neurosurgical outpatient clinic 3 months postoperatively and in this context receive additional follow-up examinations (V3). Patients who agreed to be contacted via telephone finally undergo a telephone assessment of their cognitive and functional status 1 year following surgery (V4). A synopsis of the visit plan is provided in Table 1. Recruitment is planned to be completed by December 2019. The last in-hospital follow-up visit is accordingly scheduled for March 2020, and the last telephone interview is anticipated for December 2020.

**Table 1:** Summary of the recruitment process and visit plan according to the Standard Protocol Items: Recommendations for Interventional Trials checklist.

Event				Study period								
	Enrollment	Preoperative	Intraoperative	Postoperative					3-month follow-up		1-year follow-up	
		–7d±7	0	1d	2d	3d	4d	etc		90d±14		365d±14
Eligibility screen	x											
Informed consent	x											
Demographic data		x										
Medical history		x								x		x
Cognitive testing		x								x		x
Quality of life		x								x		
Activities of daily living		x								x		
Bispectral index monitoring			x									
Vital parameters			x									
Delirium		x		x	x	x	*x*	*x*				
Medication		x	x	x	x	x	x	x		x		x
Pain		x		x	x	x	x	x				
Mobilization				x	x	x	x	x				
sMRI/re-fMRI^a^		x								x		
Cerebrovascular ultrasound		x										
Inflammatory markers		x	x	x	x							
Neural injury markers		x	x	x	x							
Brain-derived neurotropic factor polymorphism		x										

^a^sMRI/rs-fMRI: structural magnetic resonance imaging/resting-state functional magnetic resonance imaging.

### Routine Surgical Procedures

Patients included in this study suffer from degenerative spinal diseases including cervical disc herniation and stenosis, thoracical and lumbar stenosis, and degenerative instability. All patients are enrolled in elective spinal surgical procedures without an anticipated dural opening and with a minimum scheduled operative time of 60 minutes. All procedures are performed by standard neurosurgical guidelines. The operation is always performed by an experienced spine surgeon. The patients are optimally positioned on the operating table. All patients are operated on in prone position without compression of the abdomen by using proper positioning cushions. Each patient is covered with a thermal blanket throughout the operation. All operations are performed with the help of an operating microscope and a mobile x-ray device. Typical procedures include anterior cervical discectomy and fusion, posterior cervical decompression and fusion, multisegmental thoracical and lumbar decompression, and standard and complex multilevel spinal fusion.

### Routine Anesthetic Procedures

The preoperative period before the induction of anesthesia is in accordance with international standards for elective interventions. Food is withheld for a minimum of 6 hours and water for 2 hours before anesthesia starts. Oral premedication is performed with midazolam (0.1 mg/kg) depending on individual levels of preoperative excitement. After placement of a peripheral intravenous line (18- or 20-gauge catheter), anesthesia is induced by intravenous injection of sufentanyl (0.3-0.6 mg/kg) and propofol (1.5-2.5 mg/kg). Muscular relaxation is achieved with intravenous injection of cisatracurium (1.5 mg/kg). Anesthesia is maintained by a balanced anesthesia with sevoflurane. The target range chosen was 0.8 to 1.0 minimum alveolar concentration. Adequate anesthetic depth is verified via continuous monitoring of the bispectral index and real-time electroencephalography waveforms along the scalp. Estimated insensitive fluid losses are replaced isovolemic by intravenous infusion of blood isotonic electrolyte solution without lactate. A convective air warming system is used to keep the body temperature constant and normothermic. Patients are endotracheally intubated and mechanically ventilated (pressure-controlled ventilation, FiO_2_ 0.4-0.6) at a rate of 10 to 18 per minute and a positive end-expiratory pressure of 5 to 10 cm H_2_O. Tidal volume is adjusted individually on the basis of the end-tidal carbon dioxide (capnography) monitoring or blood gas analysis and the measured PaCO_2_.

Continuous recording of vital parameters includes 5-lead electrocardiography, pulse oximetry (SpO_2_), and noninvasive blood pressure measurement. Individual patients receive an arterial catheter placed in the radial artery depending on their preoperative risk profile to enable close monitoring of hemodynamics and arterial blood gas. Hypotensive situations are managed through fluid challenges and continuous medication with norepinephrine. Recovery from anesthesia was monitored in the PACU.

### Primary Outcome Measure

This study’s primary end points are duration of surgery and incidence of delirium. The hypotheses is that the duration of surgery would predict POD incidence. POD is expected to develop within 72 hours following surgery and screening is performed every 8 hours within this period in every patient using the validated Nursing Delirium Screening Scale (Nu-DESC) [7,38]. Morning and day shift screenings are performed by trained physicians during workdays, other screenings are done by trained nurses. In this study, positive screening results require confirmation by DSM-5 criteria applied by a trained physician to further increase diagnostic specificity [6]. Training of all personnel involved in the study was conducted by a neurologist with expertise in neurocritical care and ample research experience in the field. Sufficient screening performance was guaranteed at the end of the training.

### Secondary Outcome Measures

POD severity is evaluated using the Confusion Assessment Method (CAM) scoring system severity scale [39]. Subsyndromal delirium includes Nu-DESC ratings greater than zero that do not fulfill criteria for delirium. Chart-based POD screening is used to complement POD screening beyond the Nu-DESC screening period to estimate the overall in-hospital POD incidence [40].

Preoperative and postoperative cognitive abilities are evaluated at V0 and V3 using the Consortium to Establish a Registry for Alzheimer’s Disease Plus (CERAD-Plus) test battery and multiple-choice Mehrfach-Wortschatz-Intelligenztest type B (MWT-B) word test [41,42]. The CERAD-Plus includes assessments of orientation, visual naming, phonematic speed, semantic fluency, verbal episodic memory (encoding, error control, recall, discriminability), nonverbal episodic memory (encoding, recall), visuoconstruction abilities, attention, and executive speed and functions. MWT-B results reflect the general intellectual level.

Systemic inflammation, neuroinflammation, and neuronal injury are assessed with blood samples taken at V0, V1 (immediately after surgery in the PACU), and the first two days of V2 (ie, the first and second postoperative day). Systemic inflammation is characterized by white blood cell count, C-reactive protein, interleukins, and tumor necrosis factor alpha among others that are considered to contribute to the pathogenesis of delirium [15,43-45]. Markers of neuroinflammation and neuronal injury include glial fibrillary acidic protein, neuron specific enolase, and neurofilament levels [46-48]. Neopterin and malondialdehyde levels are established surrogate markers of oxidative (neuronal) stress [49,50]. Given the increasingly recognized role of genetic predisposition for neuronal plasticity, preoperative analysis of brain-derived neurotropic factor polymorphism is intended [51].

Patient-reported quality of life is assessed at V0 and V3 through the 36-item Short Form Health Survey and the Patient Records and Outcome Management Information System 29-item profile (PROMIS-29) [52,53]. Patients’ relatives are furthermore handed a proxy version of the PROMIS-29 to evaluate agreements of self- and proxy-reported quality of life regarding individual domains (PROMIS-29 proxy). Proxy reports are a valuable tool to assess patient outcome when cognitive impairment impedes self-report, yet no study previously evaluated if changes of quality of life following surgery are similarly rated by patients and their proxies [54]. Additional patient-related outcome measures include preoperative levels and postoperative changes of frailty as assessed by the Groningen frailty indicator, neck or low back pain–related disability using the Oswestry Disability Index, and anxiety and depression rated by the Hospital Anxiety and Depression Scale [55-57].

sMRI and rs-fMRI have become methods of choice to investigate neuronal correlates of pathology-related cognitive decline in delirium [58]. While there is a promising prospect for electroencephalography biomarkers to facilitate decision making in clinical situations and investigate neurophysiological changes during an episode of delirium, the spatial resolution of MRI enables the detailed investigation of brain structures and network interactions associated with the risk for POD and mechanisms, possibly preventable, leading to POCD and long-term cognitive impairment [13,59].

A recent retrospective analysis found that hemodynamic stenoses of the cerebral vasculature may predict the incidence of POD in spine surgery [60]. This study includes a prospective evaluation of this hypothesis and includes an evaluation of arterial pulsatility that was suggested as an amply available biomarker of cognitive reserve capacity [61].

### Sample Size Calculation and Statistical Methods

The primary hypothesis of this study is that the duration of surgery is a continuous predictor for POD in a binary logistic regression model, which has not been previously tested in a prospective study. Five studies performed preliminary evaluations of this relationship treating duration of surgery as a categorical variable and secondary end point. They reported mean delirium incidences of 14% for durations of surgery less than 180 minutes, 33% for 180 to 300 minutes, and 48% for surgeries lasting longer than 300 minutes [31-35]. We extend on these previous findings by using a binary logistic regression model that provides the intriguing perspective to estimate how the odds of becoming delirious change with every minute of surgery. We used a well-established simulation-based approach to estimate an adequate sample size to test our hypothesis [62]. The simulation used a representative population of surgical patients based on information from the hospital’s clinical information system, which included duration, type, and frequency of spine surgeries performed by the Department of Neurosurgery in 2016. Samples were randomly drawn from this population and included in repeated study simulations while iteratively increasing sample sizes. This process continued until 80% of simulations run for a given sample size yielded significant regression coefficients in a 2-tailed Wald test at a 5% alpha level. This approach yielded that 182 patients need to be tested so that the power to reject the null hypothesis is 80%. Anticipating a dropout rate of 10%, we plan to enroll 200 patients in this study. Before testing real data, compliance with assumptions of a binary regression analysis needs to be confirmed, including normal distribution of the data and homoscedasticity of residuals.

Secondary end points will be analyzed using appropriate summary measures depending on the distribution of data. Categorical data will be presented as absolute and relative frequencies. Continuous data will be presented as mean or median values with 95% confidence intervals. Global tests will be performed using analysis of variance for categorial data; binary and continuous data will be analyzed using generalized linear models with a suitable link function. Post hoc tests will be performed using Student *t* tests for normally distributed data, Wilcoxon signed-rank test for paired observations, or Mann-Whitney *U* test for unpaired observations. Categorical values will be compared using χ2 or McNemar. A *P* value of <.05 is denoted statistically significant. Corrections for multiple comparisons and alpha error accumulation will be performed. Statistical analysis will be performed using ‎SPSS Statistics 25 (IBM Corp) and MATLAB 2018a (The MathWorks Inc).

MRI analysis will include quantification of brain atrophy through estimations of pre- versus postoperative changes of tissue volumes. To assess the impact of cortical atrophy, brain grey matter volume will be included as an additional covariate in statistical analyses [63]. Preoperative extent and postoperative changes of white matter lesions will be quantified using the age-related white matter changes score [64]. Resting-state analyses will be conducted as previously published and particularly include the default mode network (DMN), task-positive network (TPN), salience, and dorsal attention network [13,65,66]. Regression analyses will be used to correlate network changes with alterations in domains of cognitive dysfunction.

## Results

Recruitment began in April 2018, and the study is currently enrolling patients. Data collection is expected to be finished by April 2020. This study does not receive funding from third party organizations but is supported through research budgets of involved departments. This approach was chosen to expeditiously establish a status quo supporting applications for subsequent interventional trials since the burden of POD significantly impacts clinical routine.

First results of primary end point evaluations are expected between June and July 2020. If the primary hypothesis turns out to be true (ie, duration of surgery is a predictor of POD), funding for an interventional trial will be applied for by the third quarter of 2020 and, if funding is granted, a corresponding trial to be started in 2021.

## Discussion

### Significance of This Study

This is the first study to prospectively evaluate risk factors for POD and POCD in spine surgery including comprehensive pre- and postoperative assessments of cognitive function, markers of systemic and neuroinflammation, metabolomics, cerebral vasculature, and structural and functional neuroimaging. There are no other ongoing registered studies with a similar focus [67]. The few prospective studies that evaluated risk factors and mechanisms of POD in the context of spine surgery were already discussed [31-35], however neither of the studies assessed associations of POD and POCD, which is required to disentangle pathways that promote either one or both postoperative cognitive disorders. Available retrospective studies do not resolve this issue given diagnostic inaccuracies [36,40]. Yet identification of possibly common mechanisms underlying POD and POCD would be a major step toward defining effective interventional strategies early in or even before the postoperative period, including the adaptation of surgical strategies to individual risk profiles [37]. Despite the exciting prospect for the application of possible findings from this study, there are important methodological and conceptual issues that require close attention concerning data acquisition, analysis, and interpretation.

### Diagnostic Challenges to Identify Delirium

Accurate diagnosis of POD is a major concern in all studies in the field. While diagnosing the patient using DSM-5 criteria applied by a trained specialist (eg, psychiatrist, neurologist, intensivist) is considered the method of choice, this approach is impractical in clinical routine and challenging even in study environments given the high prevalence of delirium and its fluctuating character that requires multiple assessments per day [6,68,69]. The use of screening tools, which are time efficient and can be applied by trained nurses or physicians, is hence an important step toward timely diagnosis and effective treatment of delirious patients [27,70]. A recent review of established delirium screening tools found psychometric properties to be best for the Nu-DESC and CAM, and both tools are recommended to be used by the European Society of Anesthesiology guideline on POD [70,71]. This study uses the Nu-DESC since the CAM was recently shown to be difficult to implement in practice and the Nu-DESC can be performed in less than 2 minutes and is suitable for screening by trained nurses [71-73]. Interrater reliability is not a concern using the Nu-DESC since it was reported to be substantial to excellent [71].

In order to achieve a balanced trade-off between feasibility and accuracy of diagnostic tools, we chose a combination of methods for the detection of POD regarding our primary end point. Screening for POD is performed using the Nu-DESC with a cutoff of 2 points, which provides a sensitivity and specificity of about 80% [71]. Lack of specificity is counterbalanced by subsequent confirmation of positive screening results by DSM-5 criteria [6]. While this strict approach may miss subsyndromal and mild cases of POD, we argue that it will provide robust results that are not susceptible to confounding variance introduced by cases of marginal delirium. In this context, it is important to note that current diagnostic criteria are based on phenotypes and do not reflect neurobiological endotypes, which inevitably includes the possibility that none of the available diagnostic methods will sufficiently discriminate POD endotypes from variants of physiological brain states or altered brain states of other causes [37,74,75]. Given this uncertainty, we will run secondary analyses on subsyndromal cases of POD based on Nu-DESC screening and chart-review and evaluate whether associated pathophysiological changes are continuous with endotypes of full POD.

### Contribution of Anesthesia to Neuronal Injury

Anesthesia is considered one of the major contributors to the development of POD and POCD and therefore requires close attention in every study in the field [76,77]. It is well established that the cumulative dose of anesthetics applied during surgery and the depth of sedation are modifiable risk factors for perioperative brain injury [78]. This study therefore includes continuous bispectral index monitoring for depth of anesthesia, which allows retrospective adjustment of the statistical model for confounding variance [79]. Possible mechanisms underlying nocuous effects of anesthetics include disruption of neuronal oscillations, importantly those associated with amyloid cleavage [80], induction of tau hyperphosphorylation [81], initiation of apoptotic cell-death pathways via caspase activation [82], and disruption of cholinergic transmission regulating microglia activity [83,84]. While these mechanisms were identified using single anesthetics, there is no proven benefit from using one drug over another (eg, sevoflurane or propofol) on the incidence of POD [85,86]. In the context of this preliminary evidence, we chose to standardize the anesthetic procedure using the same drugs in all patients unless the regimen needs to be changed for medical reasons (eg, due to allergies or contraindications).

### Role of Inflammatory Pathways

Investigating the role of mediators of systemic and neuroinflammation has become one of the cornerstones of POD and POCD research [37]. Research in animal models brought about exciting results, including upregulation of several inflammatory pathways and decreased neuronal plasticity in hippocampal regions while cortical regions were generally spared, which is in line with cognitive deficits observed in humans [14,15,30,87,88]. This motivated studies in humans that assessed the association of markers of inflammation with POD and POCD, yet findings were ambiguous. While some studies reported that systemic levels of interleukins, particularly interleukin-6, and C-reactive protein were predictors of brain injury, delirium, and subsequent cognitive impairment [44,89], others did not find similar associations [44,90,91]. Possible reasons for this discrepancy are that some studies included cases of intensive care unit (ICU) delirium, concentrations of markers of inflammation vary substantially between types of surgery [30,92], and neuroinflammatory effects seem to depend on the extent of preexisting neurodegeneration, which was rarely controlled for [14,93]. Another unresolved issue is how systemic and neuroinflammation interact to cause brain injury [84]. Several possible mechanisms were studied in animal models and include passive diffusion through leaky blood brain barrier [94], carrier-mediated transport [95], and de novo central production mediated through vagal afferents [96,97]. While opening of the blood barrier induced by anesthesia is an intriguing and prevailing explanation, cerebrospinal fluid levels and serum concentration of markers of inflammation are not correlated, suggesting additional involvement of other mechanisms that remain to be elucidated [47,98].

### Structural and Functional Imaging

Studying the pathophysiology of POD and POCD using MRI provides numerous opportunities to asses brain structure and function. Previous studies investigating sMRI changes found that preoperative white matter hyperintensities (WMH) were predictors of POD [99-101]. These studies, however, evaluated patients undergoing cardiac surgery or being treated in ICU, which limits their generalizability. As outlined above, cognitive trajectories in cardiac surgery can be expected to differ from other conditions given their unique hemodynamic situation that possibly affects cerebrovascular autoregulation [7]. Development of ICU delirium is associated with several risk factors that are rarely present in patients following spine surgery such as continuous sedation, ventilation, noisy environment, sleep deprivation, compromised hemodynamics, and repeated painful invasive procedures, all of which limit the interpretation of WMH as an independent risk factor [20,102]. In support of this limitation, Cavallari et al [103] examined WMH as a risk factor in a surgical population that mainly comprised orthopedic patients not treated in ICU and found no significant association with delirium. A recent review concluded that prospective studies are needed to resolve current uncertainties regarding the significance of structural abnormalities, particularly vascular abnormalities, in sMRI [104]. The situation is similar concerning the role of preexisting cortical atrophy on the risk of developing POD. Some studies reported that generalized or focal (temporal lobe, limbic system) grey matter atrophy increases the risk for delirium while others did not find this association [105,106]. A recent review interpreted differences in structural imaging to be mainly due to the focus on cardiac surgery and ICU patients, who are difficult to generalize [58]. Our study provides several potential benefits regarding mentioned limitations. We focus on a population less confounded by critical illness and also include pre- and postoperative imaging to overcome variance in the general population that limits cross-sectional comparisons to controls. We expect that these benefits and concomitant evaluations of cognitive and inflammatory profiles will help elucidate the role of sMRI changes for POD and POCD.

There are no studies that performed fMRI before surgery to identify brain network properties that predispose for the development of POD and POCD [58]. This is surprising given the broad acceptance of models that consider cognitive resilience a relevant protective mechanism and that fMRI is the method of choice to investigate neurobiological substrates underlying resilience [107-109]. This study aims to fill this gap by correlating functional data with perioperative cognitive profiles. The combination of pre- and postoperative rs-fMRI will furthermore help to disentangle brain networks that are affected by the surgical procedure and lead to sequel cognitive deficits [74]. There are currently only a few studies that provide cross-sectional data and allow for a hypothesis of involved networks including a loss of anticorrelation between the TPN and DMN, decreased DMN functional connectivity, reduced functional network integration and efficiency, and decreased functional connectivity between the posterior cingulate and superior frontal gyrus [13,110-112].

### Investigation of Perioperative Cognitive Function

The association between POD and POCD is an ongoing matter of debate [22]. While POD may accelerate the trajectory of cognitive decline, it is also possible that POD is a marker of rapid cognitive decline but does not accelerate it or that both conditions are unrelated [113]. Recent consensus statements suggest studies in the field should include investigations of both POD and POCD to elucidate their relationship and disentangle shared mechanisms [37,70]. Cognitive testing should comprise pre- and postoperative assessments to account for baseline differences, examine a broad spectrum of cognitive domains, and account for ceiling effects in good performers and floor effects in bad performers [114,115]. This study uses the MWT-B, which allows for adjustment for baseline intelligence. The CERAD-Plus test battery enables repeated measurements of cognitive abilities in multiple domains, and normative age-, education-, and gender-specific databases are available [41].

## Appendix

Multimedia Appendix 1 Standard Protocol Items: Recommendations for Interventional Trials checklist.

## Abbreviations

CAM: Confusion Assessment Method
CERAD-Plus: Consortium to Establish a Registry for Alzheimer’s Disease Plus
CONFESS: Cognitive Dysfunction Following Elective Spine Surgery in Elderly Patients
DMN: default mode network
DSM-5: Diagnostic and Statistical Manual of Mental Disorders, Fifth Edition
ICU: intensive care unit
MWT-B: Mehrfach-Wortschatz-Intelligenztest type B
Nu-DESC: Nursing Delirium Screening Scale
PACU: postanesthesia care unit
POCD: postoperative cognitive dysfunction
POD: postoperative delirium
PROMIS-29: Patient Records and Outcome Management Information System
rs-fMRI: resting-state functional magnetic resonance imaging
sMRI: structural magnetic resonance imaging
SPIRIT: Standard Protocol Items: Recommendations for Interventional Trials
TPN: task-positive network
WMH: white matter hyperintensity
