# Correction: Evaluating Mechanisms of Postoperative Delirium and Cognitive Dysfunction Following Elective Spine Surgery in Elderly Patients (CONFESS): Protocol for a Prospective Observational Trial

**DOI:** 10.2196/18469

**Published:** 2020-02-28

**Authors:** Jonas Müller, Stephan Nowak, Antje Vogelgesang, Bettina von Sarnowski, Eiko Rathmann, Sein Schmidt, Sebastian Rehberg, Taras Usichenko, Harry Kertscho, Klaus Hahnenkamp, Agnes Flöel, Henry W S Schroeder, Jan-Uwe Müller, Robert Fleischmann

**Affiliations:** 1 Department of Neurosurgery University Medicine Greifswald Greifswald Germany; 2 Department of Neurology University Medicine Greifswald Greifswald Germany; 3 Department of Radiology University Medicine Greifswald Greifswald Germany; 4 Clinical Research Unit Charité Campus Mitte Berlin Institute of Health Berlin Germany; 5 Department of Anesthesiology Protestant Hospital of the Bethel Foundation Bielefeld Germany; 6 Department of Anesthesiology University Medicine Greifswald Greifswald Germany; 7 Center for Neurodegenerative Diseases Greifswald/Rostock Germany

In the original published paper “Evaluating Mechanisms of Postoperative Delirium and Cognitive Dysfunction Following Elective Spine Surgery in Elderly Patients (CONFESS): Protocol for a Prospective Observational Trial” (JMIR Res Protoc 2020;9(2):e15488), published on February 13, 2020, there was an error in the order of authors: Robert Fleischmann was listed as the second author. The correct order of the authors is provided above, with Robert Fleischmann named last on the author list.

The correction was made in the online version of the paper on the JMIR website on February 14, 2020. Because this change was made after submission to PubMed, PubMed Central, and other full-text repositories, the corrected article has also been resubmitted to those repositories.

